# Specimen Orientation by Marking the Peripheral End: (Potential) Clinical Advantages in Prostate Biopsy

**DOI:** 10.1155/2011/270403

**Published:** 2011-07-27

**Authors:** Andrea Benedetto Galosi, Giovanni Muzzonigro, Vito Lacetera, Roberta Mazzucchelli

**Affiliations:** ^1^Institute of Urology, Azienda Ospedaliero Universitaria, United Hospitals, Torrette, 60126 Ancona, Italy; ^2^Section of Pathological Anatomy, School of Medicine, Marche Polytechnic University and Azienda Ospedaliero Universitaria, United Hospitals, 60126 Ancona, Italy

## Abstract

The aim of this paper is to identify advantages that could be obtained by orientation of the biopsy specimen using the marking technique. We reviewed our experience (4,500 cases) and the published literature. The peripheral (proximal) end of the fresh specimen is marked with ink soon after needle delivering in a few minutes. It is performed easily in association with pre-embedding method. Five potential clinical advantages were identified: (1) tumor localization, (2) atypical lesions localization and planning rebiopsy strategy, (3) planning surgical strategy, (4) selection criteria for focal therapy and active surveillance, and (5) cost reduction. Peripheral end marking is low cost, easy and reproducible. It drives several potential advantages in cancer diagnosis or isolated atypical lesions, in particular, spatial localization within the biopsy (transition versus peripheral zone, anterior versus posterior, subcapsular versus intraparenchima, and extraprostatic extension) should be easy and reliable. We can add a new pathological parameter: pathological orientation or biopsy polarity.

## 1. Introduction

PSA-driven diagnosis of Pca leads to overdiagnosis and overtreatment in part of the patient population [1]. With the advancement of active surveillance and image-guided focal therapies (brachytherapy, cryotherapy, and high-intensity focused ultrasound) as well as nerve-sparing radical surgery, the anatomical location of cancer at biopsy has become important and assumes a prominent role in treatment planning [2].

Several attempts [3, 4] have been made to improve the preoperative topographic distribution of prostate cancer in terms of number of positive cores, laterality, area of sampling (apex, mid, base), or anterior versus posterior gland. Even 3-dimensional prostate mapping based on transperineal saturation biopsy has been proposed to guide (focal or total) treatment strategy [5, 6].

Specimen orientation by marking the peripheral (proximal) end of the biopsy specimen may have several clinical advantages in order to improve the anatomical location of the cancer. Since transrectal ultrasound biopsy is the worldwide used technique for Pca diagnosis, marking the peripheral end of the core biopsy could easily identify the subcapsular tissue of the peripheral zone just close to the ultrasound probe. Only very few published papers describe the pros and cons of the marking technique [7–10].

Hypothetically, one could have four clinical scenarios if the marking of the peripheral (proximal) end of the biopsy is performed and reported by pathologist: (1) Pca reach the marked end, meaning that subcapsular peripheral zone is involved if transrectal biopsy has been used; (2) Pca is not in contact with the marked end, thus normal tissue is present in the subcapsular area, and the distance could be measured in each specimen; (3) both ends of the specimen are free of cancer, which could be interpreted as intragland cancer or tangential sampling of larger cancer located laterally to biopsy track; (4) Pca lies in the noninked end of the specimen (distal or anterior), which can be interpreted as anterior cancer. 

This hypothetical information could also be applied to isolated atypical lesions (or atipical small acinar proliferation, ASAP) detected on initial biopsy. Using marking techniques, we add more details on the anatomical location of ASAP that could be useful in the sampling strategy in case of repeat biopsy. 

The aim of this study is to identify all advantages that could be obtained by orientation of the biopsy specimen using the marking technique. We reviewed our experience and the published literature.

## 2. Methods

A MEDLINE search using keywords and MeSH term (“Prostatic Neoplasms/pathology” (Mesh) AND “Biopsy” (Mesh) AND “Staining and Labeling” (Mesh) OR “quality” OR “marking” OR “inking” (all fields)) was performed for entries between 01-01-1966 to 03-03-2011. 93 articles were retrieved from the search. In order to evaluate all papers dedicated to tissue marking techniques on prostate biopsy (PB) specimens, we selected the 35 most relevant articles based on title and abstract. Furthermore we reviewed our prostate biopsy database composed by 4,500 cases, most of them using the pre-embedding technique plus inking the peripheral end of PB. We described our technique in previous reports [11, 12]. Therefore, results are based on literature search and our experience.

### 2.1. Technique of Marking Biopsy Specimen

**Figure 1:**
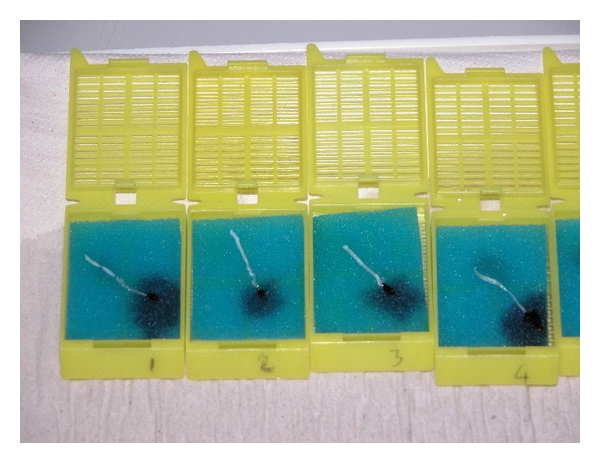
Fresh specimens of PB pre-embedded in tissue cassettes. The peripheral end of each biopsy was marked using black ink.

The marking technique can be applied to pre-embedded specimens by urologists, radiologists, or nurses in few minutes just before formalin fixation (Figure 1). The marking technique cannot be applied to free-floating specimens in formalin vials [13].

**Figure 2:**
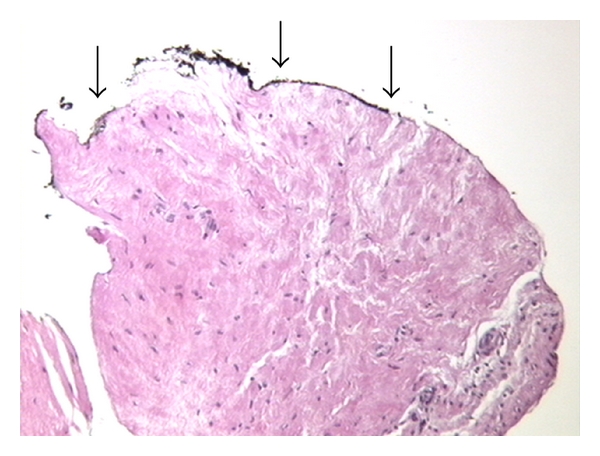
The inked end was always recognized at pathological analysis using microscope.

The proximal end of the fresh biopsy specimen is marked with ink (usually black ink) on the bench soon after needle delivering. Then the specimen is placed on nylon mesh (or sponges) and then covered with another nylon mesh according to the pre-embedding methods of prostate needle biopsy specimens described by Rogatsch et al. [13]. The specimen is closed and stretched between 2 nylon meshes and placed in a tissue-labelled cassette. One or two biopsy specimens are collected in the same cassette identified by site and prostate lobe. Afterwards the tissue cassettes were submitted in containers filled with 10% buffered formalin to obtain complete specimen fixation and dehydration. After fixation, all tissue cores were embedded in paraffin blocks and then sectioned. The inked end was always easily recognized at the pathological analysis, also in our experience (Figure 2). We called the original pre-embedding technique “sandwich technique” with the addition of few modifications [11, 12].

1. Nylon meshes (sponges) must be squeezed in saline solution to have fully soaked sponges (without air bubbles). This is very important preliminary step to biopsy specimen release, since it allows subsequent formalin diffusion and specimen fixation. If air bubbles were within the mesh, they could prevent formalin diffusion. Therefore only soaked sponge with saline solution allows complete specimen fixation and dehydration, without loss of ink. Furthermore, any potential dangerous exposure (patient or nurses) with formalin solution (contact or exhalation) is avoided using saline solution.
2. Biopsy specimens are delivered from the needle directly on nylon mesh, in order to avoid any manipulation, or changes in orientation, or loss of fragmented cores.
3. The procedure is sterile because meshes and cassettes can be treated in sterilization centre or prepacked.

Other authors [14] described that cassettes were soaked in a glass full of Bouin solution for 1 second before formalin to allow an ink fixation on the tissue. Our experience revealed that this technique was not needed and more time consuming. 

Many leading genitourinary pathologists and guidelines [15, 16] recommend the pre-embedding technique, which is considered the best one for processing and submission of PB specimens. The multipack container kits [17] are technically more complex and costly [18]; however, in 2 clinical randomized studies [19, 20] and one experimental study [21], a decreased rate of equivocal diagnosis (atypical glands and ASAP).

## 3. Results

Marking technique is performed easily on the fresh specimen in association with pre-embedding method of prostate biopsy. The inked PB end was always recognized at pathological analysis by pathologists using microscope. Five potential clinical advantages were identified using prostate biopsy specimen orientation by marking the peripheral end. We review and discuss each advantage separately.

### 3.1. Tumor Localization

Prognostic information based on biopsy other than diagnosis and grading are to quantify tumor amount within the biopsy and to identify the location of “index lesion” [22]. 

Given that Pca involves the gland in 3 distinctive ways, with different clinical implications. Specifically, (1) Pca can involve the PZ through multiple or single Pca foci originating in different prostate zones; (2) Pca can involve the TZ only through multiple or single Pca foci; (3) through peripheral zone Pca, which extends into the TZ. 

**Figure 3:**
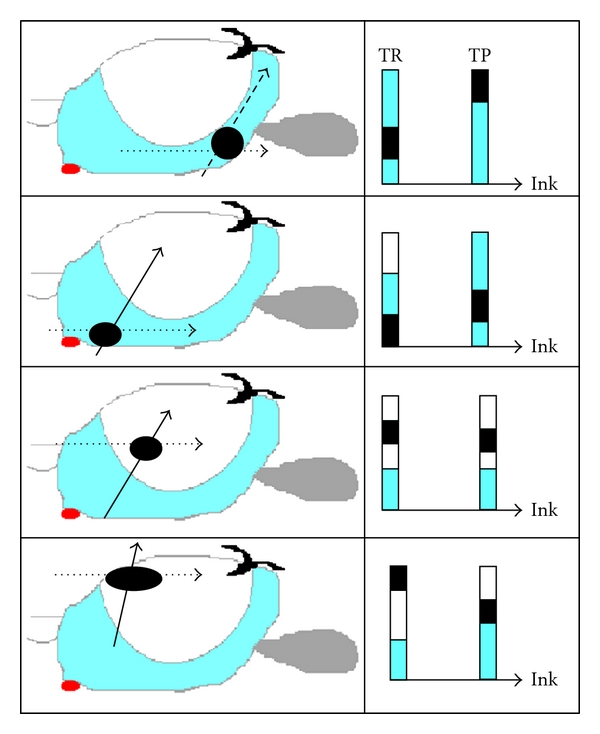
Simulation of biopsy histology corresponding to 4 different cancer foci (black circle) and normal tissue (light blue = PZ, white = TZ) in the prostate gland (longitudinal view) using 2 needle biopsy tracks: transrectal (TR; arrows) and transperineal (TP; dotted arrows). Ink = inked peripheral core end.

Specimen orientation by marking the peripheral (proximal) end could help in distinguishing these subgroups respectively (Figure 3): in the first subgroup, Pca harbours in the marked proximal end, therefore Pca involves the posterior subcapsular peripheral zone; in the second subgroup, Pca lies only in the noninked end of one or more specimens (distal), that could be interpreted as anterior zone or TZ cancer; finally the third subgroup, Pca lies in the whole specimen or both the ends of one or more specimens, that could be interpreted as PZ Pca, which extends into the TZ. 

Using the marking technique, we can add a new pathological parameter: the cancer location within the biopsy specimen.

#### 3.1.1. Anterior (TZ) versus Posterior (PZ) Cancer

Based on biopsy pathologist's report, the distinction between transition or peripheral cancer is not easy, unless the whole specimen is involved by cancer or the only positive biopsy is the TZ while all negative cores were taken from the PZ. 

**Figure 4:**
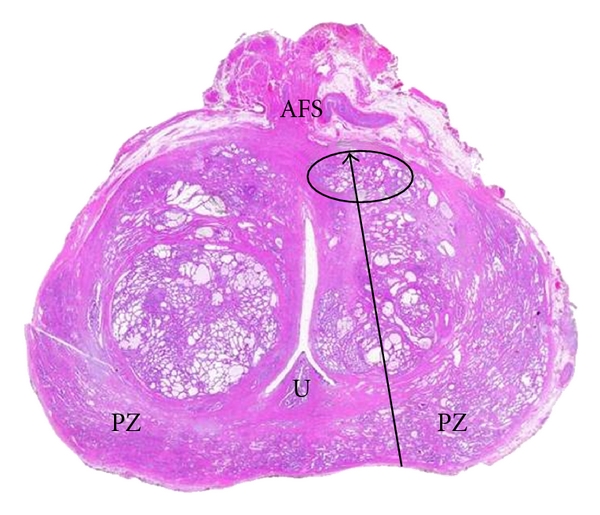
Transverse section of the whole prostate on histology after nerve-sparing surgery. The black circle shows the anterior cancer location. Anterior fibromuscular stroma (AFS), urethra (U). Biopsy track (arrow) in the anterior zone.

Inking the peripheral end of biopsy, the posterior or anterior cancer location should be easy and reliable. In particular when Pca touches the noninked end (distal), that could be interpreted as anterior cancer located in the subcapsular area (Figure 4) [23]. This pathological feature could have potential clinical advantage in the surgical management. Ponholzer et al. showed that men, with at least one core with cancer at the inked peripheral margin, had a 3.1-fold (95% CI: 1.1–9.9) increased risk for locally advanced tumour stage in the final surgical specimen [7].

Peripheral cancer has a poor prognostic outcome compared with transizion zone cancer [24], as Erbersdobler et al. [25] showed in cases matched for cancer volume. Although the distinction between subcapsular or anterior gland lesions on surgical whole specimen has been showen to have prognostic relevance, it remains unreliable on the biopsy specimen due to lacking of the specimen orientation. Therefore marking technique improves distinction between anterior and posterior Pca.

#### 3.1.2. Subcapsular versus Non-Subcapsular Cancer

**Figure 5:**
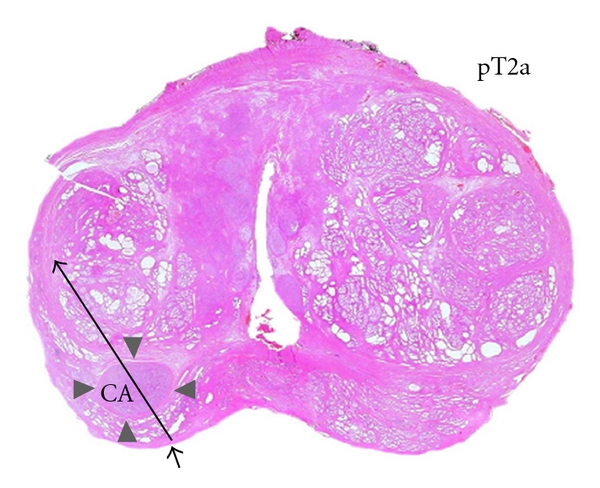
Transverse section of the whole prostate on histology showing peripheral cancer (black circle) and biopsy track (arrow). The cancer (CA) does not reach the capsule or the inked peripheral end (arrowhead) of biopsy specimen.

The pathology report may describe the distance or contact with the inked margins in cases of intraprostatic cancer (Figure 5). Pca is not in contact with the marked end, which means normal tissue is present in the subcapsular area, and the distance could be measured in each specimen.

The mean core biopsy length is 14.5 mm measured on histology slide using a needle sample notch of 18 mm (MaxiCore Bard) in our experience [11, 12] and 14.1 mm in transperineal approach using standard Tru-Cut needle [26]. The distance from cancer to the inked peripheral end (pericapsular tissue) can be measured on slides. The distance depends on the degrees of the of incidence angle of the needle on the prostate. This angle is composed by the needle penetration axis and posterior capsule axis, which ranges 15°–89° (mean 30°) adopting transrectal approach.

#### 3.1.3. Extraprostatic Cancer

Initially, the positive predictive value of an individual positive core for the location of extracapsular extension was considered not sufficient to guide the surgical decision to spare or excise a neurovascular bundle [27]. Therefore, the clinical information provided by individually labelled (cancer site itself) was previously stated not useful and not convenient to justify the increased associated costs [18]. Surprisingly, when Ponholzer et al. [7] considered the cancer location within the core (cancer located at the peripheral end of the specimen), they showed a significant correlation to pT3/pT4 stage (*P* = 0.04) in multivariate analyses of 100 cases. 

The marking technique could be useful to biopsy orientation distinguishing which end of the PB contains nonprostate tissue: nerve fibres, seminal vesicle, striated muscle or adipose tissue, Cowper gland, and fibrous capsule. All these tissue may be involved by Pca. Intraprostatic nerves (large or small fibres) are located in all subcapsular areas, therefore orientation by marking one biopsy end helps in distinction from anterior versus posterior perineural invasion. Soft tissues are rarely sampled by biopsy; however, if the tumor invasion of soft tissue has been described in the pathology report, this defines extraprostatic invasion (T3). The posterior or anterior location of extraprostatic extension based on clinical parameters and specimen marking should be easy and reliable.

The distinction of posterior or anterior invasion has importance also for radiation therapist in order to improve local cancer control, given that imaging techniques are sometimes unreliable to detect early T3.

#### 3.1.4. Application to Transperineal Biopsy

Using the transperineal approach, the needle biopsy track follows a longitudinal axis. The marking technique helps to distinguish proximal/apical (outer or perineal location) from distal/base (inner or cranial location). The advantages that we report for transrectal approach can be considered the same for transperineal.

### 3.2. Atypical Lesions (or ASAP) Localization, Planning Rebiopsy Strategy, and PIN

Hypothetically, one could have four clinical scenarios if marking the peripheral end of the biopsy has been reported after ASAP diagnosis.

1. ASAP touches the marked proximal end. Therefore atypic glands are located in the subcapsular peripheral zone, thus missed cancer could be located more proximally to the point at which the biopsy needle was fired. Clinical implication in repeated biopsy is that the needle should be activated just before insertion into the prostate capsule in order to sample all of the subcapsular area.
2. ASAP is not in contact with the marked proximal end or the distal end. That could be interpreted as tangential sampling of underlying cancer located laterally to the initial biopsy track: in particular when 2 ASAP foci are observed in the same specimen, the tangential sampling of lateral cancer should be suspected. Since ASAP has been interpreted [28–30] as tangential sampling of the underling cancer, additional biopsy should be taken also laterally to original ASAP track. Additional biopsy should be taken at the margins of the ASAP location according the transverse axis, far lateral and medial to former biopsy track, and according the longitudinal axis, proximal or distal to former biopsy track.
3. ASAP lies in the noninked end of the specimen (distal), which could be interpreted as failed sampling of anterior cancer. Therefore atypic glands are located in the far anterior zone, thus missed cancer could be located more anteriorly to the point at which the biopsy needle is sampled. Clinical implication in repeated biopsy is that the needle should be inserted 1-2 cm deeper in order to sample all the anterior area including the subcapsular area according to gland anatomy.

These advantages must be considered as potential, since no case series have never tested the usefulness of the marking technique. Cancer detection rates on repeat biopsy for ASAP using an extended core biopsy scheme, the cancer detection rate remained as high as 36% to 59.1% [28] on first repeat biopsy and 16% on second repeat biopsy [29]. Because most cancers were found in the same region as the ASAP on repeat biopsy, and because 20% to 45% of cancers can be found outside the area of ASAP, a systematic Rebiopsy of the prostate is recommended by Canadian guidelines with additional targeted cores [30, 31]. 

Different PB techniques were used to minimize false-negative biopsies in repeat biopsy populations, in our experience and daily practice we use this information in addition to other clinical and pathologic features. Saturation biopsy may be considered in high-risk cases (e.g., rising PSA, abnormal DRE, persistent ASAP) with at least 2 previous negative extended biopsies [15]. The incidence of prostate cancer at the second and third biopsy using saturation biopsy scheme versus 18-core set was 22.6% versus 10.9% (**P** = 0.02) and 6.2% versus 0% (**P** = 0.01), respectively [32, 33].

As we described above for Pca and ASAP, we may detect high-grade PIN location, transition, or peripheral zone PIN based on marking technique. Clinical relevance of this information remains to be addressed by research studies.

### 3.3. Planning Surgical Strategy

In the era of extended biopsy schemes (10–18 cores), which are now the standard of care for detection of prostate cancer, the location of extracapsular extension on prostatectomy specimen correlated well with a positive biopsy site in 70% patients [10]. Preoperative reliable cancer map based on extended biopsy could be useful to reduce positive surgical margins. In fact, aims of surgery are improving control and reduce postoperative erectile dysfunction using intrafascial (pericapsular) gland dissection leaving of few mm of extraprostatic tissue or any tissue on the surgical specimen.

Previously, Rogatsch et al. [34] evaluated the rectal margin (inked) of each core biopsy and the distance from cancer to the inked rectal end, in a selected screening population using 10-core biopsy. They showed that the predictive value of an individual positive apical biopsy was only 28.8% for predicting surgical margin positivity. They concluded that the value of preoperative individually labeled cores and rectal end inking were limited. Results were incomplete because they did not analyse individually all labeled specimens. In fact, PB of the mid, anterior, and base of the prostate were placed together in a tissue cassette whereas specimens from the apex and transition zone were placed in separate cassettes. 

Initially, Walsh [35] noted that surgical positive margin at the apex occurs during release of the dorsal vein complex and striated sphincter and not during nerve-sparing procedure. Recently, Nielsen et al. [36] modified initial poit of view introducing a link between apical dissection and nerve-sparing. In fact the high anterior release of the levator fascia in open radical retropubic prostatectomy provided excellent oncological results (1.3% positive margin) and was associated with improved postoperative sexual function (93% versus 77%). The proper selection criteria was based on Tsutzuki nomogram and cancer extent on apical biopsies [37]. The probability of side-specific extraprostatic extension was based on prostate needle biopsy pathology. They were able to predict with 90% accuracy which men would be ideal candidates for nerve-sparing surgery. 

We suggest that the multiple measures of carcinoma extent (volume) in the biopsy, tumor grade associated with individually labeled biopsy, as well as cancer at the inked peripheral end (or distance) may be the best means of predicting the risk of extracapsular disease and/or a positive surgical margin. 

Furthermore, tumor spread in prostate needle biopsy has become critical in the subsequent management of salvage treatment (surgery or cryoablation) for recurrent prostate carcinoma after radiotherapy. Mapping distribution of cancer based on biopsy is important to assess tumor spread, and it is essential for planning the salvage cryosurgery. The marking technique has a value to determine the level of depth of tumour (peripheral or periurethral, posterior or anterior) and planning of salvage cryoablation [38].

### 3.4. Selection Criteria for Focal Therapy (FT) and Active Surveillance (AS)

The anatomical cancer localization has gained more clinical and prognostic relevance in focal treatment planning. Identification of index lesion: grade, anatomical extension (size, shape, side, and zone) has gained importance in focal treatment strategy. We believe that Pca orientation within each biopsy specimen could add useful information in this clinical setting. 

In the selection of patients for FT, the transperineal saturation biopsy using the brachitherapy grid has been considered the best method to have three-dimension pathological mapping and to select patient to focal therapy [39]. Despite the requirement for general anaesthesia and a potential increased urinary retention rate, novel transperineal mapping schemes, when employing a brachytherapy grid template, allow for more accurate sampling of the entire gland. The remit of prostate biopsy now lies beyond pure diagnostics and has become an essential tool for determining the optimal therapeutic approach [39, 40]. The biopsy orientation and exact location of cancer within the specimen could be useful to depict anatomical location of Pca foci and to guide FT.

In the selection of patient for AS, the cancer location within the biopsy has not yet been considered a selection criterion. However, a different cancer location within the gland could not have the same natural history or the same success rate of therapeutic options: an example is given by subcapsular cancer or located at the edge of the gland: (1) early extraprostatic invasion has been observed in small volume Pca arising in the subcapsular tissue [41], (2) positive surgical margins have been reported in 1.5–38% Pca located at the edge of the apical/anterior even if intraprostatic (pT2). Therefore AS in small cancer located at the edge of the gland could have a negative impact on radical treatment.

## 4. Cost Reduction

The cost of histopathologic evaluation is based on number of individually labeled specimen containers. Submitting biopsy cores individually raises the cost of pathologic evaluation significantly while important prognostic information is lost when the samples are bundled into fewer containers. Marking technique is an easy method to reduce cut costs and specimen identifications. By reducing the number of specimen containers from 12 to 6, including 2 cores in each cassette, the potential savings may be in hundreds of million per year. 

Firoozi et al. showed for the first time that marking tissue-labeling is a cost-effective manner in 452 cases, while maintaining ability to glean important prognostic information from each core [10].

This tissue-labeling technique was successfully applied by Scattoni et al. in 617 patients [14]. The 24 cores were put on sponge tissue in 7 different sandwich cassettes and individually inked (Figure 1) with different colors to mark the site from which they were collected. Each single core was individually marked (black, blue, green, and orange ink). They were able recognized to each specimen separately according to anatomical sampling (lateral, subcapsular, sextant, and transition zone cores) besides 3 or 4 biopsy specimens were collected in the same cassette. 

Tissue-labeling protocol did not increase the procedure time or introduce any tissue artifacts.

## 5. Discussion

Our review supports marking the distal end of PB, since it allows spatial specimen orientation. Specimen orientation by ink marking is simple, reproducible, and does not require sophisticated technical aids. It takes approximately 2 min for the nurse to ink the cores at the peripheral end. It takes only few minutes for the incorporation into the final pathology report. It can be obtained without any patient discomfort or risk. Any tissue artefact was observed.

Furthermore the technique allows single specimen identification even if 2 or more cores are embedded in the same cassettes using different colours. Thus marking technique reduces costs while maintaining ability to glean important prognostic information from each core. 

We showed the clinical implications in repeated strategy biopsy based on relationship between inked end and atypical lesion: site, depth of needle sampling, and lateral sampling adjusted in each case according tangential sampling of main cancer foci. 

We reviewed that marking technique has several potential advantages for urologist and pathologist, but the scientific evidence in favour has been supported by expert opinion and small number of clinical studies derived from published literature. The pathological and anatomical correlation between biopsy and surgical specimen could be considered as good evidence to support this ancillary procedure. However, we defined clinical advantages as “potential” because further studies are needed to confirm all those advantages. These substantial benefits outweigh the additional effort by the pathologist.

## 6. Conclusions

Using the marking technique, we can add a new pathological parameter: pathological orientation or biopsy polarity. Cancer or atypical lesions can be accurately located within the biopsy specimen and integrated to biopsy approach. It drives several potential advantages in cancer diagnosis or isolated atypical lesions, in particular spatial localization within the biopsy (transition versus peripheral zone, anterior versus posterior, and subcapsular versus intraparenchima) should be easy and reliable.

Peripheral end marking is low cost, easy, and reproducible. It may also reduce costs allowing each specimen identification and analysis.

## Abbreviations and Acronyms

AS: Active surveillance
ASAP: Atypical small acinar proliferation
FT: Focal therapy
PB: Prostate biopsy
Pca: Prostate cancer
PIN: Prostatic intraepithelial neoplasia
PZ: Peripheral zone
TRUS: Transrectal ultrasound
TZ: Transizion zone.
